# Clinical advantages of a new collaborative assistant robot (ANSUR surgical unit) in laparoscopic appendectomy and cholecystectomy

**DOI:** 10.1007/s11701-025-03102-w

**Published:** 2026-01-03

**Authors:** Toshiya Higashi, Masaki Kimura, Yuki Kumazaki, Takuya Nakashima, Kimihiro Hattori, Mana Kawajiri, Takuya Suzuki, Koya Tochii, Shuji Takiguchi, Hidetoshi Matsunami

**Affiliations:** 1 Department of Surgery, Matsunami General Hospital, Gifu, Japan; 2 Department of Gastroenterological Surgery, Nagoya City University Graduate School of Medical Sciences, Aichi Nagoya, Japan; 3 Department of Surgery, Matsunami General Hospital, 185-1 Dendai, Kasamatsu-cho, Hashima-gun, Gifu, 501-6062 Japan

**Keywords:** ANSUR surgical unit, Collaborative assistant robot, Laparoscopic appendectomy, Laparoscopic cholecystectomy

## Abstract

To evaluate the efficacy of ANSUR-assisted laparoscopic appendectomy and cholecystectomy compared with conventional laparoscopic surgery (LS). The ANSUR surgical unit (ASAHI INTECC CO., LTD., Aichi, Japan) is a new collaborative assistant robot equipped with three arms. However, no studies have reported on ANSUR, and its clinical advantages remain unclear. We retrospectively evaluated the data of 145 patients who underwent laparoscopic appendectomy and 261 patients who underwent laparoscopic cholecystectomy, in both elective and emergency settings, at our institution between January 2023 and June 2025. ANSUR-assisted laparoscopic appendectomy was performed in 30 of 145 patients, and ANSUR-assisted laparoscopic cholecystectomy in 36 of 261 patients. In laparoscopic appendectomy, the proportion of complicated appendicitis in the ANSUR-assisted LS group was significantly lower than that in the conventional LS group (p = 0.006). However, no significant differences in the perioperative outcomes were observed between the two groups. In laparoscopic cholecystectomy, the proportion of emergency ANSUR-assisted LS cases was lower than that of conventional LS (p = 0.041). The intraoperative and postoperative outcomes of ANSUR-assisted LS were comparable to those of conventional LS. No conversion to open surgery occurred in either ANSUR-assisted laparoscopic appendectomy or cholecystectomy. The median setup time—defined as the interval between ANSUR roll-in and the start of surgery—was 5 min for laparoscopic appendectomy and 8 min for laparoscopic cholecystectomy. ANSUR is a feasible, preliminary, and safe collaborative assistive robot for laparoscopic appendectomy and cholecystectomy. Additionally, ANSUR is expected to have clinical benefit in reducing healthcare manpower constrains.

**Supplementary Information:**

The online version contains supplementary material available at 10.1007/s11701-025-03102-w.

## Introduction

Advancements in surgery include cost reduction, improved patient satisfaction, enhanced clinical outcomes, and evolving surgical approaches. Surgical techniques have evolved considerably, with minimally invasive surgery (MIS) offering several advantages, such as reduced postoperative pain, fewer complications, shorter hospital stays, and faster recovery across various procedures [1]. Typical MIS procedures include thoracoscopic, laparoscopic (LS), and robot-assisted surgery (RAS). RAS has advanced significantly since 2000 and is expected to provide surgical benefits, such as three-dimensional visualization, improved dexterity, better ergonomics, and telesurgery, while retaining the perioperative advantages of LS [2]. Moreover, the routine use of RAS has increased across multiple surgical specialties, with nearly all subspecialties adopting robotic techniques [3]. However, RAS presents several challenges, including limited evidence quality, high costs, and environmental concerns [2, 4].

Two primary types of robotic systems are used in gastroenterological surgery: teleoperated systems, in which the surgeon controls the robotic arm via a console, and collaborative robotic systems, which assist and stabilize instrument manipulation [5]. Teleoperated robotic surgical systems, such as the da Vinci Surgical System (Intuitive Surgical Inc., CA, USA), are widely used worldwide [6]. In contrast, collaborative robotic systems have been studied less extensively than teleoperated systems.

**Fig. 1:**
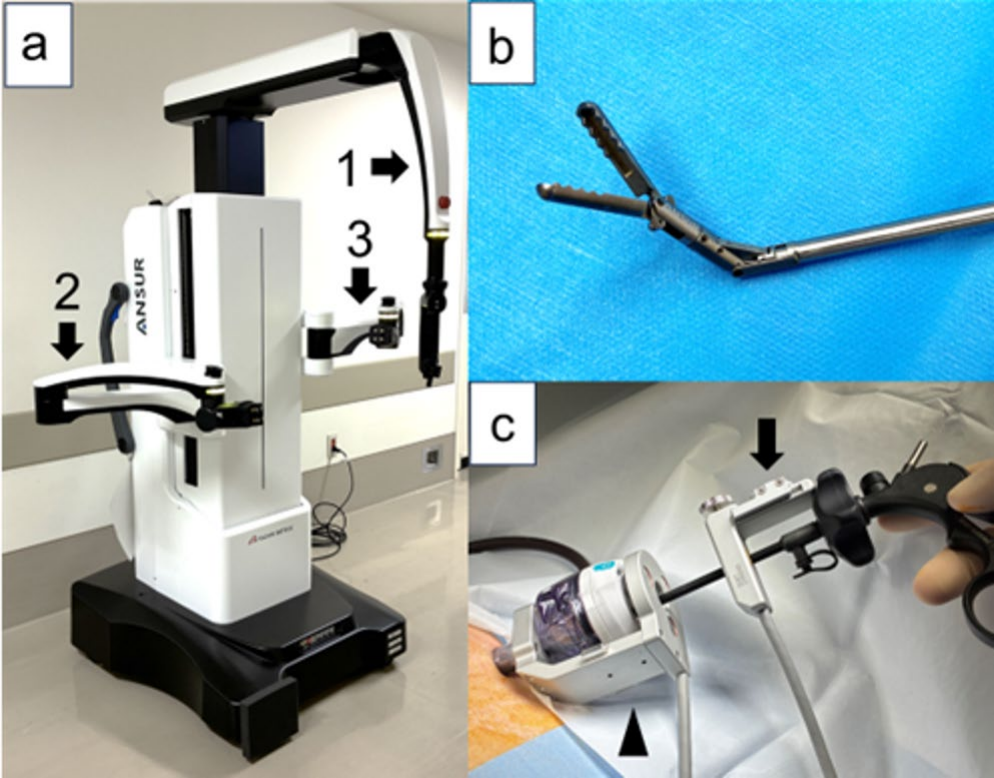
ANSUR surgical unit. (**a**) ANSUR has three arms that can be initiated and controlled by the surgeon. The arms consist of the camera arm (1), right assistant arm (2), and left assistant arm (3). (**b**) Wave forceps. (**c**) Attaching sensor (black arrow) positioned on the right-hand or left-hand side of the instrument and trocar sensor (black arrowhead)

The ANSUR surgical unit (ASAHI INTECC CO., LTD., Aichi, Japan) is a unique collaborative assistant robot that supports the operator during LS by performing assistant functions. It has three arms—one camera arm and two assistant arms (Fig. 1a)—allowing it to stabilize the camera and appropriately grasp tissue. The operator controls the camera or assistant arm—such as the wave forceps (Fig. 1b)—using a sensor attached to either side of the instrument (Fig. 1c) and a foot pedal. Consequently, ANSUR is characterized by its ability to assist surgeons in performing solo LS.

In this study, we aimed to compare the perioperative outcomes of ANSUR-assisted LS with conventional LS in appendectomy and cholecystectomy, and to evaluate its advantages, equivalence, and potential implications.

## Methods

### Patient selection

This retrospective study included patients who underwent appendectomy or cholecystectomy, either electively or emergently, at a single center (Matsunami General Hospital, Gifu, Japan) between January 2023 and June 2025. We excluded patients who underwent open procedures or other surgeries simultaneously (Supplemental Fig. 1). All patients provided informed consent through an opt-out process before enrollment in the study. This study was conducted in accordance with the ethical principles of the Declaration of Helsinki and approved by the Matsunami General Hospital Institutional Review Board (approval number: Matsu–I–Rin–658).

## Training program

The ANSUR training program was developed to include both didactic and hands-on training, incorporating tasks aimed at developing the motor and cognitive skills required to achieve competency in using ANSUR. All surgeons using ANSUR completed this training program and were fully experienced in performing conventional laparoscopic appendectomies and cholecystectomies.

## Surgical indications and procedures

The decision to perform open, elective, or emergency surgery was left solely to the operator, based on clinical judgment and patient safety. Specifically, during the early stages of introducing ANSUR-assisted LS, for laparoscopic appendectomies, we selected cases where appendiceal abscess was not suspected, regardless of whether they were emergency or elective cases. Conversely, for laparoscopic cholecystectomies, we selected elective cases. Subsequently, we performed ANSUR-assisted LS indiscriminately for both emergency and elective surgeries conducted during the day shift.

At our institution, for ANSUR-assisted laparoscopic appendectomy and cholecystectomy, the patient is placed in the supine position with the right arm tucked against the body to allow room for ANSUR.

**Fig. 2:**
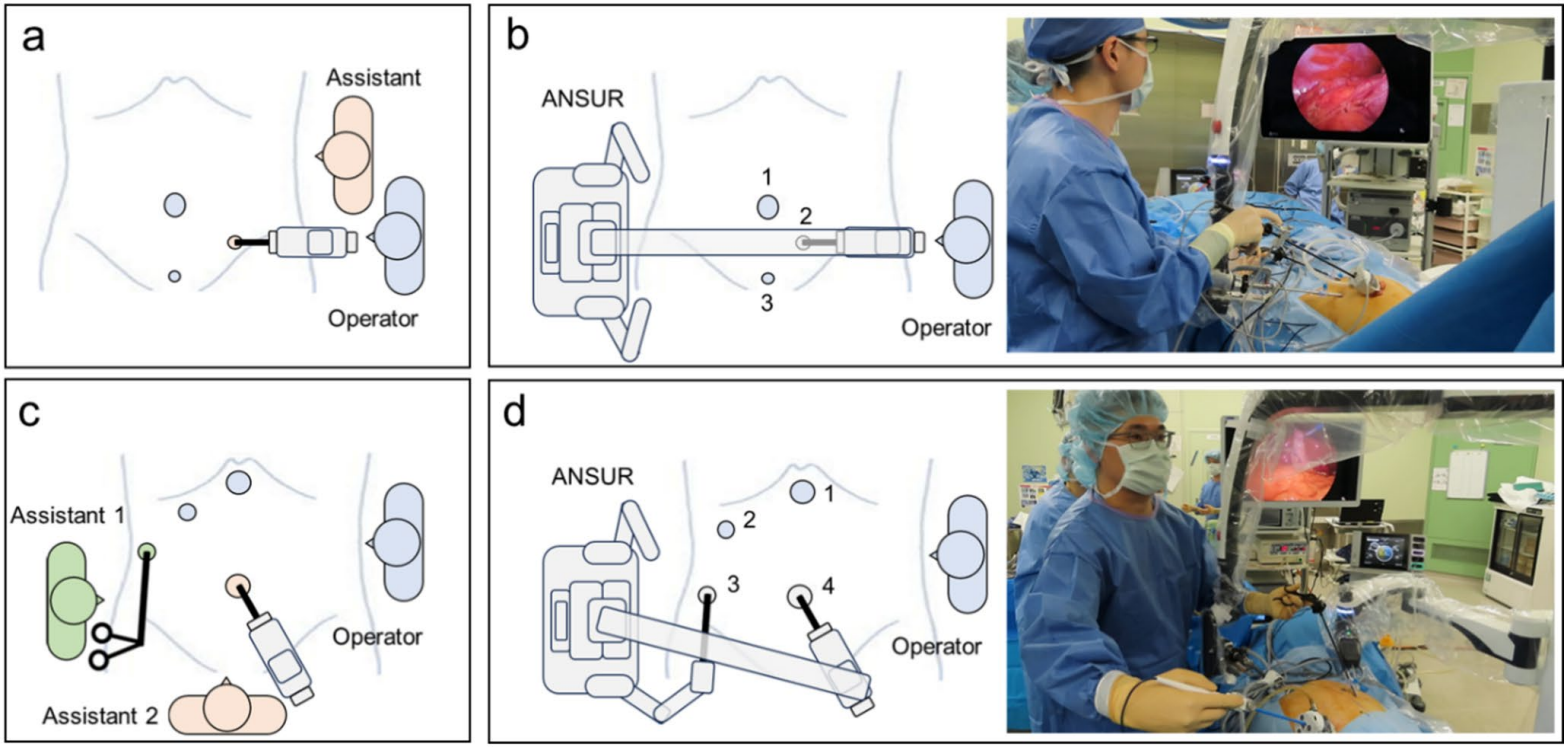
Set-up position for (**a**) conventional laparoscopic appendectomy (**b**) ANSUR-assisted laparoscopic appendectomy (**c**) conventional laparoscopic cholecystectomy (**d**) ANSUR-assisted laparoscopic cholecystectomy

Laparoscopic appendectomy requires three ports: a 12-mm port in the umbilical region and two 5-mm ports in the left lumbar and hypogastric regions, respectively (Fig. 2a, b). The monitor is placed on the right side of the operating table, adjacent to the right side of ANSUR. After adjusting the patient’s position to a head-down and left-tilted orientation and adjusting the height of the operating table, ANSUR is rolled in from the patient’s right side and docked in position. First, the camera is attached to the camera arm. Next, the camera is inserted into port 2, and its range of motion is checked. Finally, the surgeon’s port (port 1) is recognized by ANSUR. The surgeon uses the right hand for port 1 and the left hand for port 3 to perform the surgery according to routine practice.

In contrast, laparoscopic cholecystectomy requires two 12-mm ports in the umbilical and epigastric regions and two 5-mm ports in the right hypochondriac and right lumbar regions (Fig. 2c, d). The monitor is placed over the patient’s right shoulder, adjacent to the left side of ANSUR. After adjusting the patient’s position to a head-up and left-tilted orientation and adjusting the height of the operating table, ANSUR is rolled in from the patient’s right side and docked in position. First, the camera and wave forceps are attached to the camera and the right assistant arm, respectively. Next, the camera is inserted into port 4, and its range of motion is checked. Then, the wave forceps are inserted into port 3, and their range of motion is checked. Finally, the surgeon’s port (port 1) is recognized by ANSUR. The surgeon uses the right hand for port 1 and the left hand for port 2 to perform the surgery according to routine practice.

These port arrangements are similar to those used in conventional laparoscopic appendectomies and cholecystectomies. However, to prevent collisions among the patient, the surgeon, the assistant arms of ANSUR, and the external camera arm, several strategies were implemented to facilitate smooth surgical progression. In ANSUR-assisted laparoscopic appendectomy, the camera port (port 2) was shifted a few centimeters medially, particularly in patients with low body mass, to avoid collisions between the patient and the ANSUR camera arm. In ANSUR-assisted laparoscopic cholecystectomy, the port used for the operator’s left forceps (port 2) was shifted a few centimeters toward the patient’s right to avoid collision between the operator’s left arm and the ANSUR camera arm. Moreover, the assistant port (port 3) was shifted a few centimeters toward the foot side to avoid collisions between the patient and the ANSUR assistant arm.

## Evaluation of clinicopathological features and outcome measure

We investigated the patients’ demographic and clinical variables, including age, sex, body mass index, American Society of Anesthesiologists physical status, medical history, and operative and postoperative findings. Setup time was defined as the interval between the roll-in of ANSUR and the start of the surgical procedure. Severity grading of acute cholecystitis was classified according to Tokyo Guidelines 2018 [7]. Postoperative complications were defined and classified according to the Clavien–Dindo classification [8]. Information regarding the resected specimens from pathological examination was collected. Complicated appendicitis was defined as perforated or gangrenous appendicitis, with or without an intra-abdominal abscess [9]. In this study, the primary endpoint was postoperative complications, whereas the secondary endpoints were operative time. These were evaluated as additional practical factors potentially influencing the comparison between ANSUR-assisted and conventional LS.

### Statistical analysis

Patient characteristics were expressed as frequencies and percentages for categorical variables and as medians with ranges or means with standard deviations for continuous variables. Comparisons of continuous variables between the two groups were performed using the Student’s t-test or the Mann–Whitney U test. The chi-squared test was used for categorical variables. Statistical significance was defined as a *p*-value < 0.05. Statistical analyses were performed using EZR (Saitama Medical Center, Jichi Medical University, Saitama, Japan) [10], a graphical user interface for R (R Foundation for Statistical Computing, Vienna, Austria), which is a modified version of R Commander incorporating statistical functions commonly used in biostatistics.

## Results

### Patient characteristics

A total of 421 patients were initially included in the study; however, 15 were excluded because they underwent different procedures (seven cases began as open surgery, and eight laparoscopic procedures were performed simultaneously with other surgical interventions). Thus, 406 patients were included in the final analysis. Of these, 145 patients underwent laparoscopic appendectomy, and 261 underwent laparoscopic cholecystectomy (Supplemental Fig. 1).

## Comparison of ANSUR-assisted and conventional laparoscopic appendectomy

The clinicopathological characteristics and perioperative outcomes in laparoscopic appendectomy are summarized in Table 1. ANSUR-assisted LS was performed in 30 of the 145 cases. No significant differences were observed between the two groups in clinical characteristics or intraoperative and postoperative outcomes, except for diagnosis. The proportion of patients with complicated appendicitis was 23.3% in the ANSUR-assisted LS group and 42.6% in the conventional LS group, with a significantly higher proportion of complicated appendicitis in the conventional LS group (*p =* 0.006). Additionally, no conversions to open surgery were observed in either group. The median setup time for the ANSUR was 5 (range, 3–16) min. Table 2 presents a comparison of the two groups stratified by emergency and elective surgery. In elective cases, ANSUR-assisted LS resulted in fewer cases of complicated appendicitis (9.1% vs. 26.9%) and a higher proportion of other diagnoses (36.4% vs. 3.8%) compared to conventional LS (*p* = 0.024).

**Table 1:** Patients’ characteristics and perioperative outcomes in laparoscopic appendectomy

Variables	ANSUR-assisted LS	Conventional LS	*p*-value
	*n* = 30	*n* = 115	
Age (years), median (range)	50.5 (12–82)	46 (10–91)	0.702
Sex (Male/Female), n (%)	17 (56.7)/13 (43.3)	70 (60.9)/45 (39.1)	0.834
Body mass index (kg/m^2^), median (SD)	21.5 (3.2)	22.1 (3.3)	0.349
ASA PS classification (I/II/III/IV/V), n	22/7/1/0/0/0	63/40/10/1/1	0.436
History of abdominal surgery, n (%)	13 (11.3)	4 (13.3)	0.999
Emergency case, n (%)	19 (63.3)	89 (77.4)	0.181
Operating time (min), median (range)	59 (35–91)	70 (26–196)	0.138
Set-up time (min), median (range)	5 (3–16)		
Blood loss (mL), median (range)	1 (0–20)	1 (0–380)	0.623
Conversion to open surgery, n (%)	0 (0)	0 (0)	
LOHS (days), median (range)	4 (2–14)	4 (2–30)	0.114
Complications (CD grade ≥ III), n (%)	0 (0)	4 (3.5)	0.699
Diagnosis, n (%)			0.006
Complicated appendicitis	7 (23.3)	49 (42.6)	
Simple appendicitis	19 (63.3)	64 (55.7)	
Others	4 (13.3)	2 (1.7)	
Re-admission, n (%)	0 (0)	0 (0)	

LS, laparoscopic surgery; SD, standard deviation; ASA PS, American Society of Anesthesiologists’ physical status; LOHS, postoperative length of hospital stay; CD, Clavien–Dindo

**Table 2:** Patients’ characteristics and perioperative outcomes in emergency and elective laparoscopic appendectomy

	Emergency case *n* = 108				Elective case *n* = 37		
	ANSUR-assisted LS *n* = 19	Conventional LS *n* = 89	*p*-value		ANSUR-assisted LS *n* = 11	Conventional LS *n* = 26	*p*-value
Operating time (min), median (range)	63 (35–91)	70 (28–159)	0.337		58 (38–86)	70 (26–196)	0.287
Blood loss (mL), median (range)	1 (0–20)	1 (0–380)	0.411		1 (0–3)	1 (0–110)	0.691
LOHS (days), median (range)	4 (2–14)	4 (3–30)	0.185		3 (3–5)	3 (2–6)	0.859
Complications (n)			0.813				0.338
CD I					SSI (1)		
CD II	Paralytic ileus (2)	Paralytic ileus (4) SSI (2) Asthma (1)				Paralytic ileus (1)	
CD IIIa		Paralytic ileus (2)				SSI (1)	
CD IVa		VT (1)					
Diagnosis, n (%)			0.392				0.024
Complicated appendicitis	6 (31.6)	42 (47.2)			1 (9.1)	7 (26.9)	
Simple appendicitis	13 (68.4)	46 (51.7)			6 (54.5)	18 (69.2)	
Others	0 (0)	1 (1.1)			4 (36.4)	1 (3.8)	

LS, laparoscopic surgery; LOHS, postoperative length of hospital stay; CD, Clavien–Dindo; SSI, surgical site infection; VT, ventricular tachycardia

### Comparison of ANSUR-assisted and conventional laparoscopic cholecystectomy

Table 3 summarizes patients’ characteristics and perioperative outcomes for laparoscopic cholecystectomy. ANSUR-assisted LS was performed in 36 of 261 cases. The proportion of emergency surgery cases was 19.4% in ANSUR-assisted LS and 38.7% in the conventional LS group, with a significantly higher proportion in conventional LS (*p* = 0.041). ANSUR-assisted LS resulted in significantly less intraoperative blood loss (1 mL vs. 5 mL, *p* < 0.001) and shorter postoperative hospital stays (4 [range, 2–8] days vs. 4 [range, 2–27] days, *p* = 0.002). Additionally, no conversion to open surgery was noted in ANSUR-assisted LS. The median set-up time for the ANSUR was 8 (range, 3–23) min. Table 4 presents a comparison of the two groups stratified by emergency and elective surgery. In emergency cases, ANSUR-assisted LS resulted in shorter postoperative hospital stays (4.5 days vs. 7.0 days, *p* = 0.008) and lower complication rates (*p* = 0.010) compared to conventional LS. In elective cases, ANSUR-assisted LS resulted in less intraoperative blood loss compared to conventional LS (1 [range, 0–45] mL vs. 1 [range, 0–150] mL, *p =* 0.001).

**Table 3:** Patients’ characteristics and perioperative outcomes in laparoscopic cholecystectomy

Variables	ANSUR-assisted LS	Conventional LS	*p*-value
	*n* = 36	*n* = 225	
Age (years), median (range)	63.5 (35–82)	66.0 (22–91)	0.502
Sex (Male/Female), sex, n (%)	20 (55.6)/16 (44.4)	122 (54.2)/103 (45.8)	0.999
Body mass index (kg/m^2^), median (range)	23.9 (15.6–37.3)	24.0 (14.7–40.5)	0.862
ASA PS classification (I/II/III), n (%)	9 (25.0)/21 (58.3)/6 (16.7)	50 (22.2)/131 (58.2)/44 (19.6)	0.886
History of abdominal surgery, n (%)	9 (25.7)	71 (31.7)	0.606
Emergency cases, n (%)	7 (19.4)	87 (38.7)	0.041
Preoperative PTGBD, n (%)	0	4 (1.8)	0.940
Operating time (min), median (range)	91.5 (61–141)	98.0 (43–283)	0.230
Set-up time (min), median (range)	8 (3–23)		
Blood loss (mL), median (range)	1 (0–250)	5 (0–1,200)	< 0.001
Conversion to open surgery, n (%)	0 (0)	2 (0.9)	0.999
LOHS (days), median (range)	4 (2–8)	4 (2–27)	0.002
Complications (CD grade ≥ III), n (%)	1 (2.9)	17 (7.6)	0.509
Diagnosis, n (%)			0.105
Choledocholithiasis	8 (22.2)	44 (19.6)	
Acute cholecystitis	6 (16.7)	83 (36.9)	
Chronic cholecystitis	19 (52.8)	81 (36.0)	
Others	3 (8.3)	17 (7.6)	
Severity grading of acute cholecystitis (I/II/III), n (%)	2 (33.3)/4 (66.7)/0 (0)	44 (53.0)/37 (44.6)/2 (2.4)	0.100
Re-admission, n (%)	0	4 (1.8)	0.955

LS, laparoscopic surgery; ASA PS, American Society of Anesthesiologists’ physical status; PTGBD, percutaneous transhepatic gallbladder drainage; LOHS, postoperative length of hospital stay; CD, Clavien–Dindo

**Table 4:** Patients’ characteristics and perioperative outcomes in emergency and elective laparoscopic cholecystectomy

	Emergency case *n* = 94			Elective case *n* = 167		
	ANSUR-assisted LS *n* = 7	Conventional LS *n* = 87	*p*-value	ANSUR-assisted LS *n* = 29	Conventional LS *n* = 138	*p*-value
Operating time (min), median (range)	115 (68–141)	107 (61–222)	0.966	90 (61–135)	94 (43–283)	0.498
Blood loss (mL), median (range)	3 (0–250)	10 (0–1,200)	0.195	1 (0–45)	1 (0–150)	0.001
LOHS (days), median (range)	4.5 (3–5)	7.0 (3–26)	0.008	3 (2–8)	4 (2–27)	0.251
Complications (n)			0.010			0.883
CD I	SSI (1) Liver dysfunction (1)	SSI (1)		(0)	SSI (1)	
CD II	Liver dysfunction (1)	Liver dysfunction (2) Atrial fibrillation (2) Paralytic ileus (1) Postoperative bleeding (1) Pneumonia (1) Urinary tract infection (1)		(0)	Paralytic ileus (2) Drug rash (1) DGE (1) Urinary retention (1)	
CD IIIa	(0)	SSI (4) Choledocholithiasis (4) Intra-abdominal abscess (1)		SSI (1)	SSI (6) Choledocholithiasis (1) Septicemia (1)	
Diagnosis, n (%)			0.511			0.865
Choledocholithiasis	0 (0)	0 (0)		8 (27.6)	44 (31.9)	
Acute cholecystitis	6 (85.7)	81 (93.1)		0 (0)	2 (1.4)	
Chronic cholecystitis	1 (14.3)	4 (4.6)		18 (62.1)	77 (55.8)	
Others	0 (0)	2 (2.3)		3 (10.3)	15 (10.9)	

LS, laparoscopic surgery; LOHS, postoperative length of hospital stay; CD, Clavien–Dindo; SSI, surgical site infection; DGE, delayed gastric emptying

## Discussion

We demonstrated that no significant difference in surgical outcomes were observed between ANSUR-assisted laparoscopic appendectomy and the conventional method. Moreover, intraoperative and postoperative outcomes were largely equivalent between ANSUR-assisted laparoscopic cholecystectomy and the conventional method. No additional ports or conversions to open surgery were required in either ANSUR-assisted laparoscopic appendectomy or cholecystectomy. Additionally, the setup time for ANSUR was short, and no apparent adverse events occurred during ANSUR-assisted LS.

Further, we showed that the proportion of patients with complicated appendicitis in ANSUR-assisted laparoscopic appendectomy was significantly lower than that in the conventional method. Similarly, the proportion of emergency surgery cases in ANSUR-assisted laparoscopic cholecystectomy was significantly lower than that in the conventional method. Furthermore, in laparoscopic cholecystectomy, ANSUR-assisted LS resulted in significantly less intraoperative blood loss, shorter postoperative hospital stays, and a lower incidence of complications in emergency surgery. Regarding complications, the case requiring the longest hospitalization period in conventional laparoscopic cholecystectomy involved an emergency surgery for acute cholecystitis in a bedridden patient with a gastrostomy tube. Postoperatively, the patient developed delayed gastric emptying, requiring a prolonged period for his condition to stabilize through enteral nutrition via the gastrostomy tube. These results are likely attributable to selection bias and surgeon bias. During the early stages of introducing ANSUR-assisted LS, elective and less complex surgeries were selected to ensure safety, and nearly all procedures using ANSUR were performed by more experienced and highly skilled surgeons. Nevertheless, by selecting appropriate cases and having procedures performed by well-experienced surgeons, ANSUR can be considered safe and capable of achieving outcomes comparable to the conventional method.

Collaborative robotic systems in surgery began in 1994, when AESOP (Computer Motion, Santa Barbara, CA, USA) was first used clinically in 1993 and subsequently marketed in 1994 as the first surgical robot approved by the Food and Drug Administration. In gastroenterological surgery, the AESOP 3000 (Computer Motion), introduced in 1998, is a voice-controlled robotic camera holder. AESOP showed no difference in perioperative outcomes compared to human assistance in cholecystectomy and colectomy [11, 12]. However, it has been reported that in laparoscopic cholecystectomy and laparoscopic hernioplasty, AESOP 3000 results in reduced surgeon comfort, longer preparation and operation times, despite the advantage of decreased personnel requirements [13].

EndoAssist (Armstrong Healthcare Ltd., High Wycombe, United Kingdom), introduced in the late 1990 s, is a robotic camera holder activated by foot control and operated via a headset-mounted motion axis selection sensor. In laparoscopic cholecystectomy, EndoAssist has been shown to result in significantly shorter operative times compared to human assistance [14] and has also been introduced in colorectal surgery [15].

Soloassist and Soloassist II (AKTORmed, Barbing, Germany), introduced in the early-to-mid 2010 s, are robotic endoscope holders maneuvered using a joystick. Soloassist has been shown to be safe for use in cholecystectomy without increasing complications, although operating times are longer than with human assistance [16]. Soloassist II has been associated with fewer participating surgeons and reduced postoperative hospital stays following laparoscopic cholecystectomy [17]. It has also been shown to reduce operating time compared to human assistance in laparoscopic inguinal hernia repair [18] and to be as effective as human assistance in laparoscopic colorectal cancer surgery [19, 20].

ViKY (EndoControl, Grenoble, France), introduced in 2007, is a robotic laparoscope holder controlled by either a foot pedal or voice activation, providing direct vision control to the surgeon. No differences were observed between ViKY and conventional methods regarding postoperative complications or recurrence following laparoscopic inguinal hernia repair [21].

FreeHand (FreeHand Ltd., Guildford, United Kingdom), introduced in 2009, is a robotic laparoscope holder positioned alongside the patient and attached to the operating table. The operator directly controls the robotic arm using a foot pedal and a head-mounted radiofrequency communicator that responds to head movements. FreeHand has been reported to be safely used in laparoscopic appendectomy [22], colectomy [23], and other gastroenterological surgeries [24]. These studies reported that FreeHand provides surgical outcomes comparable to conventional LS, a stable image, minimized user discomfort, and reduced the need for additional personnel.

EMARO (HOGY Medical, Tokyo, Japan), introduced in 2015, is a freestanding laparoscopic camera holder controlled by infrared signals from a head attachment worn by the operator and reportedly reduces labor costs and postoperative pain [25]. Maestro (Moon Surgical SAS, Paris, France), introduced in 2022, is a two-arm robotic device that holds and assists in the positioning of laparoscopes and instruments. Maestro has reportedly been used safely in several LS procedures in gastroenterological surgery without conversion to open surgery and has resulted in a high surgeon satisfaction index [26].

ANSUR is a new type of collaborative assistant robot with three arms that enables it to hold the camera in a stable position and appropriately grasp tissue (Fig. 1a). The surgeon moves the camera and wave forceps using sensors attached to instruments on either the right- or left-hand side, along with a foot pedal. Sensors attached to the surgeon’s instruments and trocars provided feedback to each robotic arm regarding the position and movement of surgical instruments, allowing the camera and wave forceps to move to the desired position (Fig. 1c). Wave forceps are wave-shaped, double-ended grasping forceps with a 5-mm-diameter shaft that bends at the tip (Fig. 1b). Therefore, the surgeon can move the wave forceps for proper deployment of the surgical field. Regarding ANSUR’s setup, we showed that ANSUR had a short setup time and a straightforward setup procedure. Therefore, surgeons can operate the system smoothly after only limited experience. Given its characteristics, ANSUR may provide a stable field of view and facilitate operative field development, allowing surgeons to perform safer and less stressful solo surgical LS, reduce operative time, and minimize physical stress on the patient. Providing a stable surgical field during surgery improves surgical outcomes [27]. Therefore, the stable surgical field provided by ANSUR may contribute to the surgeon’s steady performance, potentially leading to the reduced intraoperative bleeding, fewer postoperative complications, and shorter hospital stays observed in this study. However, one issue with ANSUR is that by taking on an assistant role, it may eliminate the role of surgical trainees as assistants, potentially depriving them of opportunities to learn LS. From an educational perspective, similar to teleoperated robotic surgical systems, ANSUR-assisted LS offer the advantage of enabling the supervising surgeon to seamlessly switch with a surgical trainee when necessary without disrupting the surgical field. This facilitates step-by-step instruction while maintaining safety.

The primary advantage of ANSUR-assisted LS is its ability to reduce manpower requirements while maintaining applicability in both elective and emergency surgeries. Especially, ANSUR can perform the duties of both an assistant and a scope operator, taking on roles that preciously required one or two staff members. This is expected to improve the work-life balance of medical staff, including surgeons. For surgeons in particular, the time saved by using ANSUR can be allocated to ward duties, reviewing patient records, taking breaks, attending other surgeries, or performing surgeries in parallel. Moreover, ANSUR can be implemented in existing operating rooms without the need for special renovations or complex setup, allowing for flexible responses even in emergency surgeries.

Cost remains a major consideration for the adoption of new technologies. Compared to conventional LS, the operative cost of ANSUR-assisted laparoscopic appendectomy was €261 higher per operation, and that of ANSUR-assisted laparoscopic cholecystectomy was €494 higher per operation. The additional cost of ANSUR-assisted LS was calculated based on the total number of consumables (Supplemental Fig. 2). Specifically, this included the sterile covers for the body, assistant arms, and camera arm; the cost of trocars and surgical tool sensors (calculated by dividing the total cost by 30 uses); and the cost of wave forceps (calculated by dividing the total cost by 10 uses). This analysis did not include initial acquisition costs, depreciation, or service contracts for robotic and laparoscopic systems. Nevertheless, the additional costs of ANSUR-assisted laparoscopic appendectomy and cholecystectomy were lower than those reported for teleoperated robotic surgical systems in previous studies [28–30]. Therefore, ANSUR-assisted LS may offer advantages in terms of cost-effectiveness. Moreover, using ANSUR during surgery can reduce the number of medical staff required, potentially leading to savings in labor costs.

This study has some limitations. First, it was a retrospective study conducted at a single institution. Second, the number of enrolled patients, particularly those who underwent ANSUR-assisted LS, was relatively small; therefore, the findings may be biased, with limited generalizability. Third, surgeon bias was noted in this study. In ANSUR-assisted LS, almost all procedures were performed by surgeons certified by Endoscopic Surgical Skill Qualification System of the Japan Society for Endoscopic Surgery. In contrast, conventional LS procedures were performed by both certified and non-certified surgeons. However, in surgeries performed by non-certified surgeons, a certified surgeon always participated as an assistant, providing appropriate guidance when necessary to ensure surgical safety and to compensate for the inexperience of the non-certified surgeon. Therefore, a more comprehensive, large-scale, prospective study should be conducted to validate our findings. Nevertheless, to our knowledge, this was the first study to report the feasibility of a new type of collaborative assistant robot for LS.

In conclusion, although the cost of ANSUR-assisted LS was slightly higher than that of conventional LS, ANSUR can be considered a feasible, preliminary, and safe collaborative assistive robot. In addition, ANSUR is expected to alleviate surgeon shortage, improve community healthcare, and reform the way surgeons work.

## Supplementary Information

Below is the link to the electronic supplementary material.

Supplementary material 1 (DOCX 679.2 kb)

## Data Availability

Data are available upon reasonable request to the corresponding author.
