# Supplementary material for: Clinical advantages of a new collaborative assistant robot (ANSUR surgical unit) in laparoscopic appendectomy and cholecystectomy
Source: J Robot Surg. 2026 Jan 3;20(1):140. doi: 10.1007/s11701-025-03102-w (PMC12764531; doi:10.1007/s11701-025-03102-w)
Supplement: Supplementary file 1 — Supplementary material 1 (DOCX 679.2 kb) [file 11701_2025_3102_MOESM1_ESM.docx]

**Supplementary Figures:**


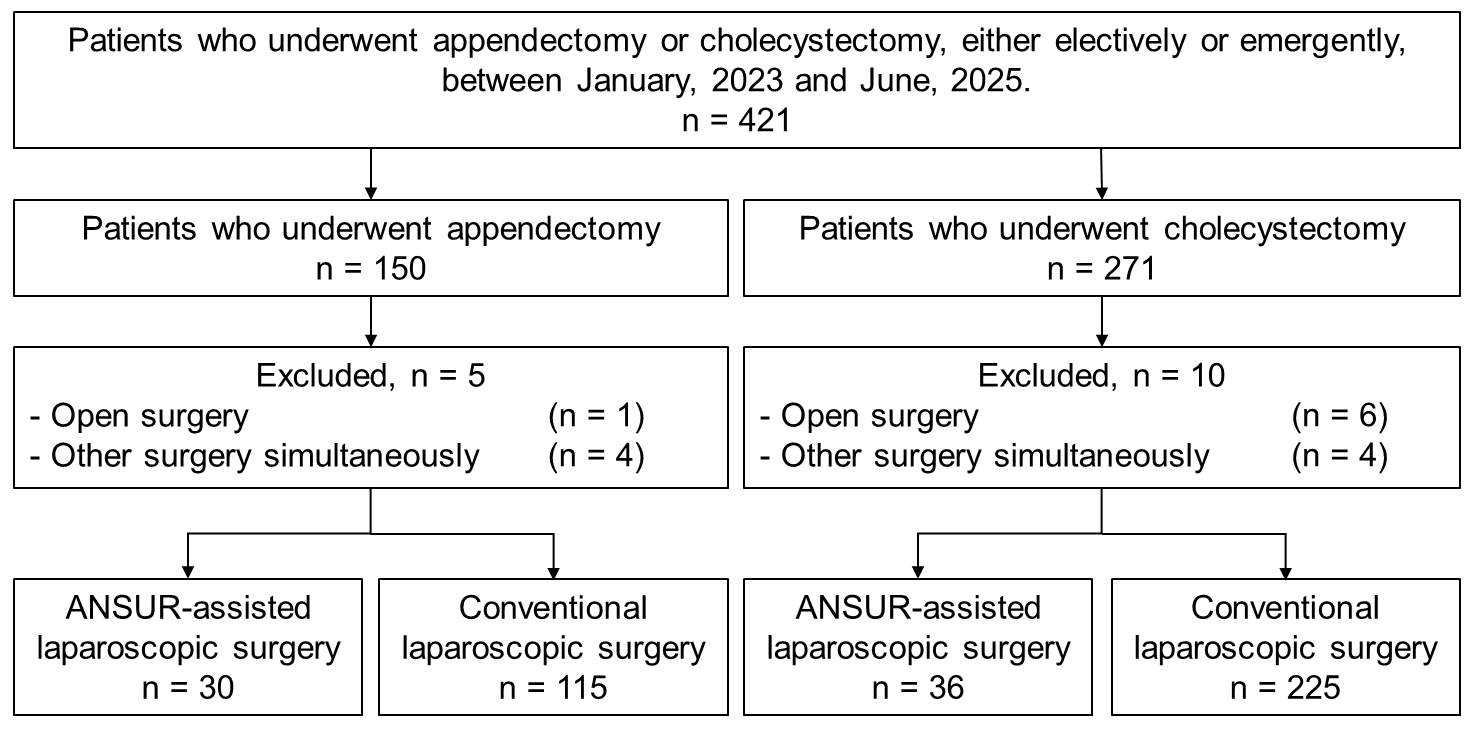


Supplemental Fig. 1 Flow chart of patient selection


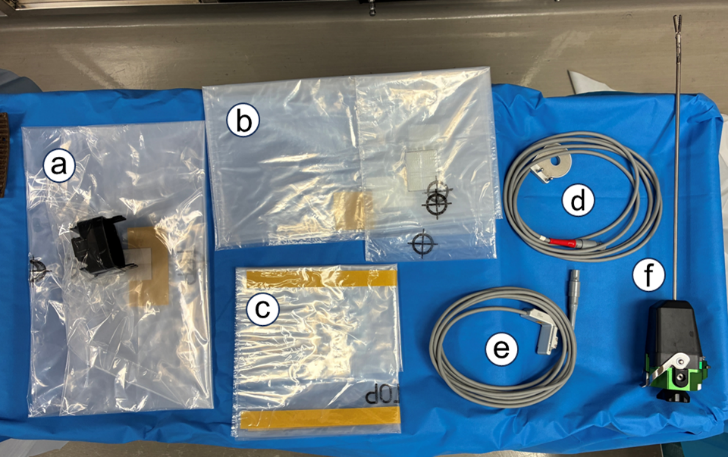


**Supplemental Fig. 2** Consumables

1. Sterile cover of the assistant arm
2. Sterile cover of the body
3. Sterile cover for camera arm
4. Trocar sensor
5. Surgical tool sensors
6. Wave Forceps
